# Supplementary material for: Predicting visual function by interpreting a neuronal wiring diagram
Source: Nature. 2024 Oct 2;634(8032):113–23. doi: 10.1038/s41586-024-07953-5 (PMC11446822; doi:10.1038/s41586-024-07953-5)
Supplement: Supplementary file 6 — CRF and ERF predictions for individual TmY4 and TmY9 cells. Analogous to Supplementary Data 3, but for TmY target types. Shown are the top four monosynaptic pathways, the strongest pathway passing through each of the top ten intermediary types (ranking from Extended Data Fig. 7), and the trisynaptic pathway Tm1–TmY–Dm3–TmY (see the section entitled Prediction of spatial normalization). [file 41586_2024_7953_MOESM6_ESM.zip › DataS4/TmY4/720575940625417360.pdf]

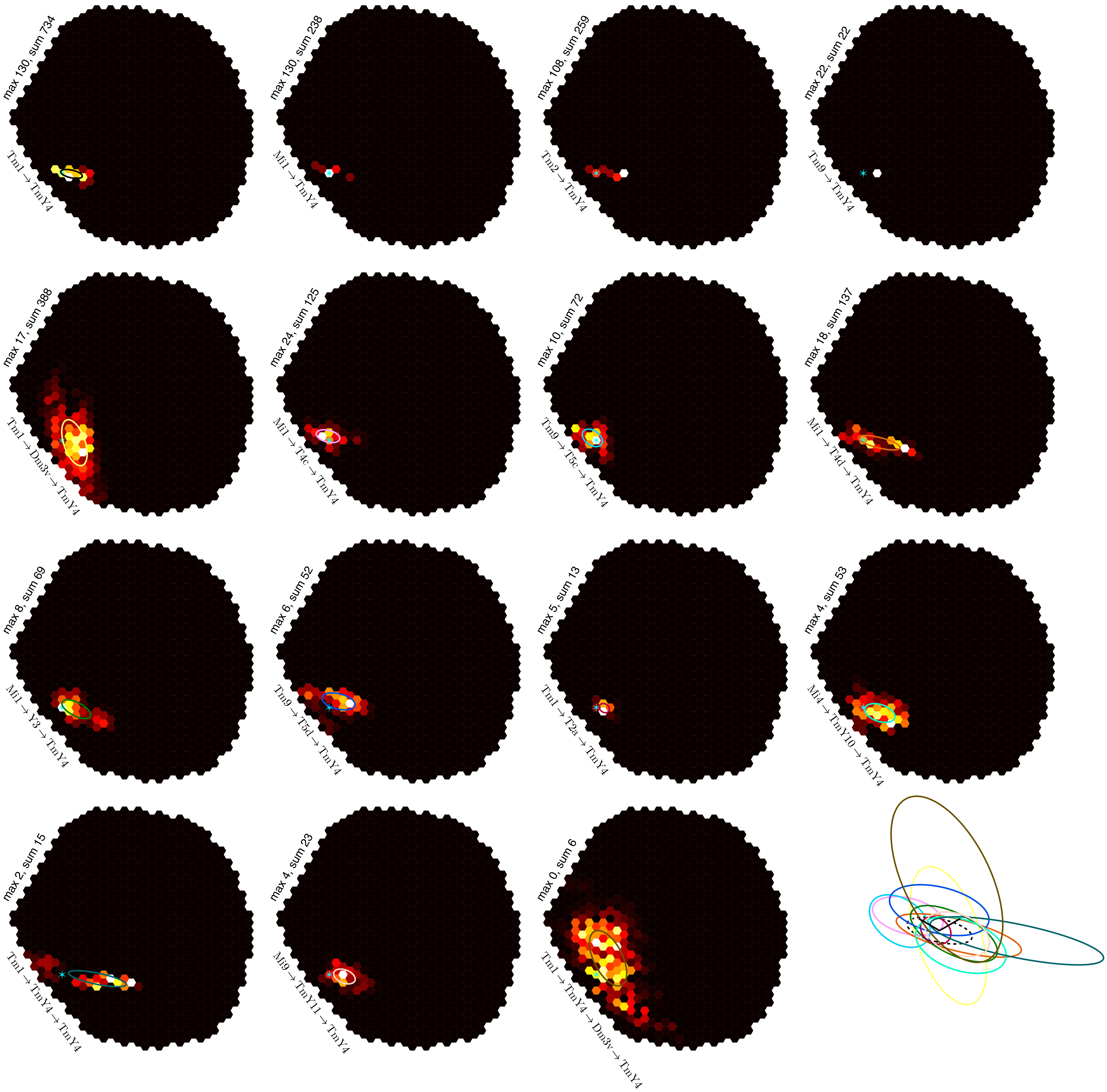

max 130, sum 734
Tm1 → TmY4
max 130, sum 238
Mi1 → TmY4
max 108, sum 259
Tm2 → TmY4
max 22, sum 22
Tm9 → TmY4
max 17, sum 388
Tm1 → Dm3v → TmY4
max 24, sum 125
Mi1 → T4c → TmY4
max 10, sum 72
Tm9 → T5c → TmY4
max 18, sum 137
Mi1 → T4d → TmY4
max 8, sum 69
Mi1 → Y3 → TmY4
max 6, sum 52
Tm9 → T5d → TmY4
max 5, sum 13
Tm1 → T2a → TmY4
max 4, sum 53
Mi4 → TmY10 → TmY4
max 2, sum 15
Tm1 → TmY4 → TmY4
max 4, sum 23
Mi9 → TmY11 → TmY4
max 0, sum 6
Tm1 → TmY4 → Dm3v → TmY4
